# Thermal Modulation of Monoamine Levels Influence Fish Stress and Welfare

**DOI:** 10.3389/fendo.2018.00717

**Published:** 2018-12-03

**Authors:** Nataly Sanhueza, Andrea Donoso, Andrea Aguilar, Rodolfo Farlora, Beatriz Carnicero, Jesús Manuel Míguez, Lluis Tort, Juan Antonio Valdes, Sebastian Boltana

**Affiliations:** ^1^Department of Oceanography, Interdisciplinary Center for Aquaculture Research, Biotechnology Center, University of Concepción, Concepción, Chile; ^2^Instituto de Biología, Facultad de Ciencias, Universidad de Valparaíso, Valparaíso, Chile; ^3^Laboratorio de Fisioloxía Animal, Departamento de Bioloxía Funcional e Ciencias da Saúde, Facultade de Bioloxía, Universidade de Vigo, Vigo, Spain; ^4^Departamento de Biología Celular, Inmunología i Fisiologia Animal, Universidad Autónoma de Barcelona, Barcelona, Spain; ^5^Facultad de Ciencias de la Vida, Departamento de Ciencias Biológicas, Universidad Andrés Bello, Santiago, Chile

**Keywords:** confinement, fish husbandry, behavior, HPI-axis, thermoregulation

## Abstract

Fish are ectotherm organisms that move through different thermal zones according to their physiological requirements and environmental availability, a behavior known as thermoregulation. Thermoregulation in ectothermic animals is influenced by their ability to effectively respond to thermal variations. While it is known that ectotherms are affected by thermal changes, it remains unknown how physiological and/or metabolic traits are impacted by modifications in the thermal environment. In captivity (land-based infrastructures or nets located in the open sea), fish are often restricted to spatially constant temperature conditions within the containment unit and cannot choose among different thermal conditions for thermoregulation. In order to understand how spatial variation of temperature may affect fish welfare and stress, we designed an experiment using either restricted or wide thermal ranges, looking for changes at hormonal and molecular levels. Also, thermal variability impact on fish behavior was measured. Our results showed that in Atlantic salmon (*Salmo salar*), a wide thermal range (ΔT 6.8°C) was associated with significant increases in monoamines hormone levels and in the expression of clock genes. Aggressive and territoriality behavior decreased, positively affecting parameters linked to welfare, such as growth and fin damage. In contrast, a restricted thermal range (ΔT 1.4°C) showed the opposite pattern in all the analyzed parameters, therefore, having detrimental effects on welfare. In conclusion, our results highlight the key role of thermal range amplitude on fish behavior and on interactions with major metabolism-regulating processes, such as hormone performance and molecular regulatory mechanisms that have positive effects on the welfare.

## Introduction

Nowadays, there is considerable public debate regarding welfare of fish kept in captivity, specifically under aquaculture conditions. This debate has also raised interest in the scientific community, leading to precise the meaning of welfare in fish through the study of their emotional and cognitive capacities and how captivity may influence these parameters. In this context, studying the dynamics of social interactions in captive fish is of high relevance for understanding the behavioral biology of a particular species as well as for optimizing its production and avoid negative impacts of culture procedures (1–3). Social interactions are crucial for the development of specific traits, which rely upon a succession of particular interactions among individuals, leading to the development of hierarchies (4). Among those interactions, the result of aggressive encounters has been described as a key factor determining the establishment of dominant or subordinated individuals (5), and for both, aggressive encounters imply an intense social stress (6, 7).

Regarding aquaculture species, salmonids from the genus *Salmo* stand out among other major commercial fish species as they develop, either in nature or captivity, social hierarchies that rely on dominance and social rank, both having important consequences in the physiology and life history of individuals (8, 9). In captive fish, it has been observed that environmental modifications drive changes at hormonal and physiological levels, impacting the social stress and welfare (10). The molecular mechanisms underlying these changes have been widely studied in both endotherms and ectotherms. Among them, circadian regulator genes, monoamine neurotransmitters like serotonin (5-HT) and dopamine (DA) and hormones such as melatonin and cortisol have been demonstrated to play an important role in the stress response and social interaction (11–13). In addition, these molecules have been widely used as welfare and stress assessment indicators, as they are involved in homeostatic mechanisms responsive to allostatic challenges and aggressive behaviors (14, 15).

It has been extensively recognized that appropriate environmental enrichment, defined as a deliberate increase in environmental complexity, with the aim to reduce maladaptive and aberrant traits in fish reared in otherwise stimuli-deprived environments, is widely used in research and culture conditions as a tool for managing fearfulness, undesirable behaviors, stress and welfare of captive animal (16). Understanding the effects of the environmental thermal enrichment in the regulation of behavior and physiology is of high relevance for the identification of the biological mechanisms associated with stress and welfare, not only for captive fish, but also in wild fish under the thermal stress conditions associated to climate change. Given the considerations mentioned above, this study assessed how thermal enrichment can improve the welfare of *Salmo salar*. In our work, the paradigm of maintaining fish at a selected fixed temperature and in contrast, allowing the fish to choose among a broad range of temperatures, thus helping fish to develop natural behavior is discussed. We propose that fish aggressive behavior associated to an estricted thermal environment is driven by a neuroendocrine and molecular response. In order to test this hypothesis, we used a *Salmo salar* model to ask the following three questions:

- Can environmental thermal enrichment improve salmon health?
- How does thermal range amplitude affect fish hormone performance and, ultimately the behavior?

## Materials and methods

### Fish husbandry and experimental conditions

**Figure 1:**
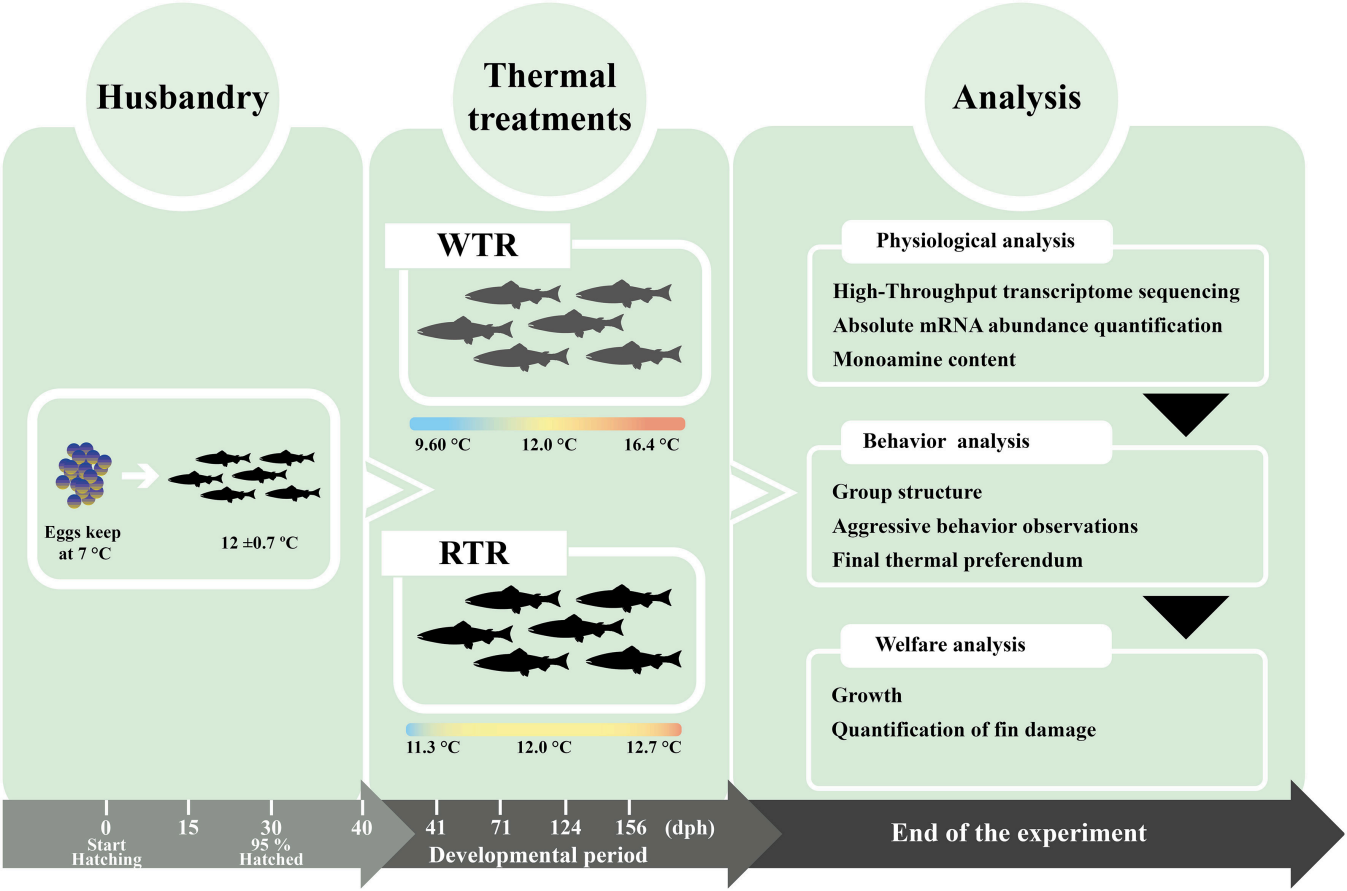
Experimental design the diagram shows the thermal set-up treatments (restricted thermal range; ΔT 1.4°C and wide thermal range; ΔT 6.8°C) for *Salmo salar* rearing over a developmental period (156 dph).

All animal experiments conformed to international animal research regulations (the British Home Office Regulations. Animal Scientific Procedures Act 1986; care guidelines, EU 2010/63) and follow the guidelines for the use of laboratory animals established by the Chilean National Commission for Scientific and Technological Research (CONICYT), authorized by the Universidad de Concepcion Institutional Animal Care and Use Committee. Thermal experiments were carried out at the ThermoFish Lab, Biotechnology Center, University of Concepcion, Concepción, Chile. Fertilized eggs (*n* = 8,000) at the eyed egg stage from *Salmo salar* were obtained from AquaGen S.A. (Melipeuco, Chile) in December 2015. After arrival, the eggs were disinfected in iodophore (100 ppm) for 15 min. followed by a wash with clean water. Hatchery conditions were used as described by Boltaña et al. (17). Fish embryos were initially maintained in a temperature-controlled room (18°C). Two recirculating freshwater systems (210 × 150 × 90 cm) were used, with each system using UV-sterilized water and a flow rate of 5 m^3^ h^−1^. Each system contained three independent tanks (60 × 140 × 70 cm). The water temperature of each tank was measured twice per day (7 ± 0.7°C). Each tank was supplied with oxygen, with a saturation between 100 and 150% throughout the entire experiment. Dissolved oxygen was also measured daily and always remained above 9 mg/L^−1^. Ammonia, nitrite and pH were measured twice per week. Total ammonia and nitrite concentrations in each tank were maintained under 0.05 and 0.01 mg L^−1^, and pH remained at 8.0 ± 0.5. A 24-h dark cycle photoperiod was used until the embryos hatched. Water temperature, pH and dissolved oxygen were measured with a Multiparameter Water Quality Meter 9829 (Hanna Instruments^Ⓡ^, Woonsocket, United States). Ammonia and nitrate / nitrite were measured with QUANTOFIX^Ⓡ^ test strips (MACHEREY-NAGEL, Dueren, Germany). Larvae were gradually acclimatized to a 12-h light: 12-h dark photoperiod cycle and after the yolk was fully absorbed (40 days post hatching, [dph]), water temperature was increased by 1°C per h until reaching the required thermal ranges. As a note, *S. salar* specimens were raised from first feeding (40 dph) to 5 months post hatching under a 12-h light: 12-h dark photoperiod to artificially reproduce the Autumn–Winter seasons, in correspondence with the annual cycle of species (18). First-feeding (f.f.) was initiated about 40 days after hatching. For the first 2 weeks after f.f., fish were fed according to appetite. Thereafter, they were fed at a daily rate corresponding to approximately 2.5% of bodyweight. Larvae were fed with biomar larvae diet (INICIO PLUS follow by PLUS 18%, Biomar, S.A., Puerto Montt, Chile) twice a day for 5 months. All experiments were performed in a temperature-controlled room (12°C). Fish were randomly assigned to two thermal treatment groups according to Boltaña et al. (19) and kept at a density of 10 kg/m^3^. Temperature gradients for both groups were established using an external water jacket system set at different temperatures. This setup provided a continuous vertical thermal gradient within the tanks, thus generating two treatment conditions (Figure 1): (1) wide thermal range (WTR), ΔT 6.8°C (Tmin 9.6°C to Tmax 16.4°C) from top to bottom, respectively, thereby mimicking a natural thermal gradient and (2) restricted thermal range (RTR), ΔT 1.4°C (Tmin 11.3°C to Tmax 12.7°C). Water temperature in the vertical column was recorded by thermal sensors located at different points within the water column (Thermocouple thermometer 53/54 II; Fluke^Ⓡ^ Corporation, Washington, United States). No significant differences were recorded in oxygen levels throughout the gradient. In the WTR, the temperature gradient was constantly maintained (i.e., 24/7). The gradient ranges were set based on (1) the natural thermal range of *S. salar*, which is between 7 and 22°C (20, 21) and (2) constant laboratory conditions that considered the most common land-based farming conditions. Eight individuals were randomly sampled per point, and tissues were stored in RNAlater^Ⓡ^ solution (Ambion, United States) at −80°C. To inspect the post morten analysis liver and kidney and gills of lived or dead fish were fixed in 10% buffered formalin for histopathological- and immune-histochemical (IHC) examination (data no shown).

### Monoamine analysis

For the hormone analysis, the brain and the whole body of three replicates per treatment (WTR and RTR) and time (41, 71, 124, and 156 dph) were snap-frozen in liquid nitrogen and conserved at −80°C. The brain and body content of melatonin, dopamine (DA), 3,4-dihydroxyphenylacetic acid (DOPAC, a major da metabolite), 5-HT, and 5-hydroxyindoleacetic acid (5-HIAA) were analyzed in triplicate by high performance liquid chromatography with electrochemical detection as previously described by Gesto et al. (22). Briefly, tissues were homogenized by ultrasonic disruption in 0.5 ml of mobile phase with the following composition: 85 mM sodium hydrogen phosphate, 0.72 mM octanosulfonic acid, 18% methanol, and adjusted to pH 3.0. Homogenates were centrifuged (16,000 × g for 10 min at room temperature) and prior to analysis supernatants were diluted 1:1 or 1:2 (supernatant/mobile phase). A 20-μl aliquot of each sample was injected into the HPLC system consisting of a Jasco PU2080 pump equipped with a Jasco AS-2057 autosampler, and an ESA Coulochem II detector (Bedford, MA, USA). The detection system included a M5011 ESA analytical cell with electrode potentials set at +20 and +300 mV, respectively. All separations were performed at room temperature at a flow rate of 8 ml/min. Acquisition and integration of chromatograms were performed by using the ChromNAV version 1.12 software (Jasco Corp.).

### RNA extraction, cDNA synthesis and mRNA abundance quantification

Random fish (*n* = 24, four per tank) were sampled for each treatment (WTR and RTR) and time (41, 71, 124, and 156 dph) and subsequently snap-frozen in liquid nitrogen and conserved at −80°C. Total RNA was extracted from brain and liver with the TRI Reagent^Ⓡ^ (0.5 mL; Sigma-Aldrich Missouri, United States) and quantified by absorbance at 260 nm. Only samples with an A260/280 ratio between 1.8 and 2.1, and an A260/230 ratio above 1.8 were used for reverse transcription. Purified RNA integrity was confirmed by agarose denaturing gel electrophoresis. cDNA was synthesized from total RNA (200 ng/μL) using the RevertAid H Minus First Strand cDNA Synthesis Kit (Fermentas, Waltham, MA, United States) according to the manufacturer's indications. RT-qPCR was performed using the StepOnePlus™ Real-Time PCR System (Applied Biosystems, Life Technologies, Carolina, United States), and each assay was run in triplicate using the Maxima SYBR Green qPCR Master Mix (2×) (Bio-Rad, Carolina, United States). cDNA used in qPCR assays was first diluted with nuclease free water (Qiagen, Hilden, Germany). Each qPCR mixture contained the SYBR Green Master Mix, 2 μl cDNA, 500 nmol/L each primer, and RNase free water to a final volume of 10 μl. Amplification was performed in triplicate on 96- well plates with the following thermal cycling conditions: initial activation for 10 min at 95°C, followed by 40 cycles of 15 s at 95°C, 30 s at 60°C, and 30 s at 72°C. A dilution series made from known concentrations of plasmid containing the PCR inserts was used to calculate absolute copy numbers for each of the genes examined (17). Real-time PCR assay was carried out in order to analyze the expression pattern in the liver of salmon for genes related to biosynthesis and function of cortisol (Steroid Acute Regulatory Proteins (*stara* and *starb*) and Glucocorticoid Receptor 1 (*gr1*) and in the brain for different clock genes (*bmal, clock, per1, cry2, aanat2, nr1d1;* see details Supplementary Table 1).

### High-throughput transcriptome sequencing: library construction and illumina sequencing

To drive this analysis the brain of nine individuals from each experimental set-up (WTR and RTR) time (14, 41, 71, 124, and 156 dph) was dissected, snap-frozen in liquid nitrogen and conserved at −80°C. RNA was individually isolated using Ribo-Pure™ Kit (Ambion^Ⓡ^, United States) according to the manufacturer's instructions. The obtained RNA was subsequently treated with DNase I (Fermentas, Massachusetts, United States) to remove genomic DNA according to the manufacturer's protocol. RNA integrity number (RIN) was evaluated through the 2200 TapeStation (Agilent technologies, California, USA) using the R6K screen tape and reagents (Agilent Technologies, California, USA). Samples with RIN ≥ 8 and 260/280 ratio ≥1.8 were used for library construction. Total RNA from each condition (RTR and WTR) and time (41, 71 124, and 156 dph) were pooled (*n* = 3 individuals by time and treatment) and quantified with Qubit^Ⓡ^ 2.0 Fluorometer (Invitrogen, California, United States). Samples pools were prepared for Illumina sequencing using KAPA Stranded mRNA-Seq Kit (KapaBiosystems, Massachusetts, United States) according to the manufacturer's instruction. Libraries were analyzed on the 2200 TapeStation (Agilent technologies, California, United States) using D1000 screen tape and reagents (Agilent Technologies, California, United States) and quantified by qPCR using the Library Quantification Kit Illumina/Universal (KapaBiosystems, Massachusetts, United States) according to the manufacturer's instructions before pooling for sequencing on MiSeq (Illumina, Inc., California, United States) platform using a run of 2 × 250 paired-end reads at the Laboratory of Biotechnology and Aquatic Genomics, Interdisciplinary Center for Aquaculture Research (INCAR), Universidad de Concepción, Chile. The raw data for each sequenced developmental stage were separately trimmed by removing adaptor sequences, low quality sequences (quality score of 0.05), and sequences with lengths <50 nucleotides, using the CLC Genomics Workbench software (Version 11.0.1, CLC Bio, Denmark). The cleaned reads from each developmental stage were used to analyze the differential expression between WTR and RTR groups, and the relative gene transcription levels for a panel composed of 28 transcripts associated with biosynthesis of monoamines and circadian clock. These sequences were used as references to map the reads of each dataset. Using the CLC Genomic Workbench software, values for the reads per kilobase per million mapped reads (RPKM) were separately calculated from the mapping sequences obtained for each developmental stage (two library replicates per stage). The settings used were a mismatch cost of 2, an insert cost of 3, a length fraction of 0.8 and a similarity fraction of 0.8. To visualize the results, a volcano graph showing differentially expressed genes between WTR and RTR groups was used (23). In addition, a hierarchical clustering of features was executed for the dataset, and a heatmap was constructed to plot significant differences in gene transcription between the developmental stages of *S. salar*.

### Gene ontology (GO-DAVID analysis) and interactome analysis

Enrichment of specific gene ontology (GO) terms among the set of probes that are specific to challenges was assessed to correlate a specific set of mRNAs within a brain. In all GO analyses, Ensembl Gene Identifiers were tested using DAVID Bioinformatics Resources (https://david.ncifcrf.gov/home.jsp), (24, 25). Enrichment of each GO term was evaluated through use of the Fisher's exact test and corrected for multiple testing with FDR [pFDR < 0.05; (26)]. We applied a Bonferroni correction to account for multiple tests performed. Each gene set comprised of at least 4 transcripts that shared the same GO biological process or annotation term. The final GO enrichment analysis was carried out with the Cytoscape Cytoscape 3.5.1. (http://www.systemsbiology.org). Topological analysis of individual and combined networks was performed with Network Analyzer, and jActiveModules 2.2 was used to analyze network characteristics (27, 28). GO analyses were conducted with the Biological Network Gene Ontology (ClueGO, version 2.0) plugin (29) used for statistical evaluation of groups of proteins with respect to the current annotations available at the Gene Ontology Consortium (http://www.geneontology.org). In addition, we conducted a complementary analysis with ClusterMaker cytoscape plugin (30), using the MCL algorithm to search protein–protein interaction network modules derived from TAP/MAS (tandem affinity purification/mass spectrometry). This approach clustered the network into modules based on PE Score to indicate the strength of the node association and given a fixed set of genes with high protein–protein affinity (interactome cluster nodes).

### Group structure and behavioral records

To analyse fish group structure, we captured images at each successive 30 min, resulting in 48 images. One group of 10 individuals of each tank (three groups for each thermal treatment) was used. The position of each fish in each image was mapped using its XY coordinates and determined by its center of mass using Image-Pro Plus 7 software (Media Cybernetics, Inc., Rockville, MD, United States). For group structure, we used three parameters to characterize each individual: the nearest neighbor distance (NND), the mean of inter-individual distances (D) and the variance of these inter-individual distances (V) as previously described by Colchen et al. (31). For each group of thermal treatment, the mean and SE of NND, D and V were calculated from 48 images. Aggressive behavior was recorded from the front and side of the tank. A total of 4 videos per tank were obtained. Ten minutes of each video recording were analyzed. Although experimental fish had been accustomed to and not disturbed by the presence of the observer, the first minute of each video was excluded to eliminate possible disquiet caused to the fish by the setting of the camera. Aggressive behavior was estimated by counting the number of aggressive acts as previously described Batzina and Karakatsouli (32) and Batzina et al. (33). Behavioral patterns observed and counted as one aggressive act were: (a) chasing without nipping or biting, (b) nipping without prior chasing, (c) biting without prior chasing, (d) chasing that ended up as nipping and (e) chasing that ended up as biting. Data refer to the whole fish group since fish were not marked and it was impossible to identify which one performed or received an attack. Group structure and behavioral records were analyzed for three consecutive days at the end of the experiment (154, 155, and 156 dph).

### Quantification of Fin damage

Digital photographs were taken at the end of the experimental period and fin damage was evaluated in every fish using the Relative Fin Index (RFI) as described by Bosakowski and Wagner (34). RFI has been suggested to allow reliable and objective measurement of the degree of fin damage (35) and it was obtained by dividing the maximum total fin length (longest fin-ray length from body) by the fork length in each individual fish. Pectoral, caudal and dorsal fins were measured and quantified using this index. Fin erosion was measured using an ordinal scale of 0, 1, 2, and 3, corresponding to no erosion (0% of fin eroded), mild erosion (1–24% of fin eroded), moderate (25–49% of fin eroded) and severe erosion (>50% of fin eroded), respectively (36, 37).

### Growth analysis

Random samples (*n* = 10 per group) were taken at 41, 71, 124, and 156 dph. Sampled fish were anesthetized with MS-222 (3-aminobenzoic acid ethyl ester; Sigma, Vienna, Austria). Body mass (W [g]) was measured using a microbalance Radwag WTB 200 (RADWAG^Ⓡ^, Radom, Poland) and digital photographs were taken for body length measurement (TL [mm])using the Image-Pro Plus 7 software (Media Cybernetics, Inc., Rockville, MD, United States). The individual data of the effect of two-thermal treatments on the weight-length relationships were plotted together in order to examine whether there was any pattern in the way that W changes correlated with TL across group. Additionally, Fulton's condition factor (K) was calculated (38). In addition, the interindividual variance of weight was calculated by computing of coefficient of variation (CV) distributions based on individuals from each thermal group. CV value was calculated for each group by dividing the standard deviation of its weight by its average group weight and present as a CV percentage.

### Final thermal preferendum

The final thermal preferendum analysis was carried out by recording the thermal behavior of fish in each experimental set-up (WTR and RTR). Three video cameras provided continuous monitoring of each tank. During the experiment, temperatures were recorded for 10 s every 15 min throughout 24 h (96 recorded events). Fish distribution inside the tank was monitored over time with video cameras and the number of fish in each compartment was counted manually from the images captured at each successive 15 min, resulting in 96 measurements per day. WTR was achieved with a mean difference in temperature of 6.8°C (Tmin 9.6°C to Tmax 16.4°C). RTR was achieved with a mean temperature of 12.0 ± 0.7°C (ΔT 1.4°C; Tmin 11.3°C to Tmax 12.7°C). All temperatures were recorded each day at the same time of the day.

### Statistical analyses

All data was tested for normality and variance homogeneity s using the Shapiro-Wilk's and Levene's test, respectively. When necessary, data was log_10_ transformed to achieve normality and homogeneity in all variances. For gene expression (absolute mRNA quantification by real-time polymerase chain reaction qPCR), monoamine content, relative fin index (RFI), total number of fish affected, quantification degree of fin damage, total length, body mass and Fulton's condition factor (K), data was analyzed using a two-way ANOVA followed by the Tukey HSD *post-hoc* test for multiple comparisons, using treatment (WTR and RTR) and sample time (41, 71, 124, and 156 dph), or fin (dorsal, perctoral and caudal) as independent variables. For aggressive behavior observations, group structure and body weight variation coefficients, the obtained data was analyzed using Student's *t*-test. Statistical analysis was undertaken using JMP 11.0.0 (SAS Institute, Cary, NC, United States) and results were considered significant when *p* < 0.05. Graphs were plotted with GraphPad PRISM v6.0 (GraphPad Software, Inc. California, United States).

## Results

### Analysis of brain transcriptomes

**Figure 2:**
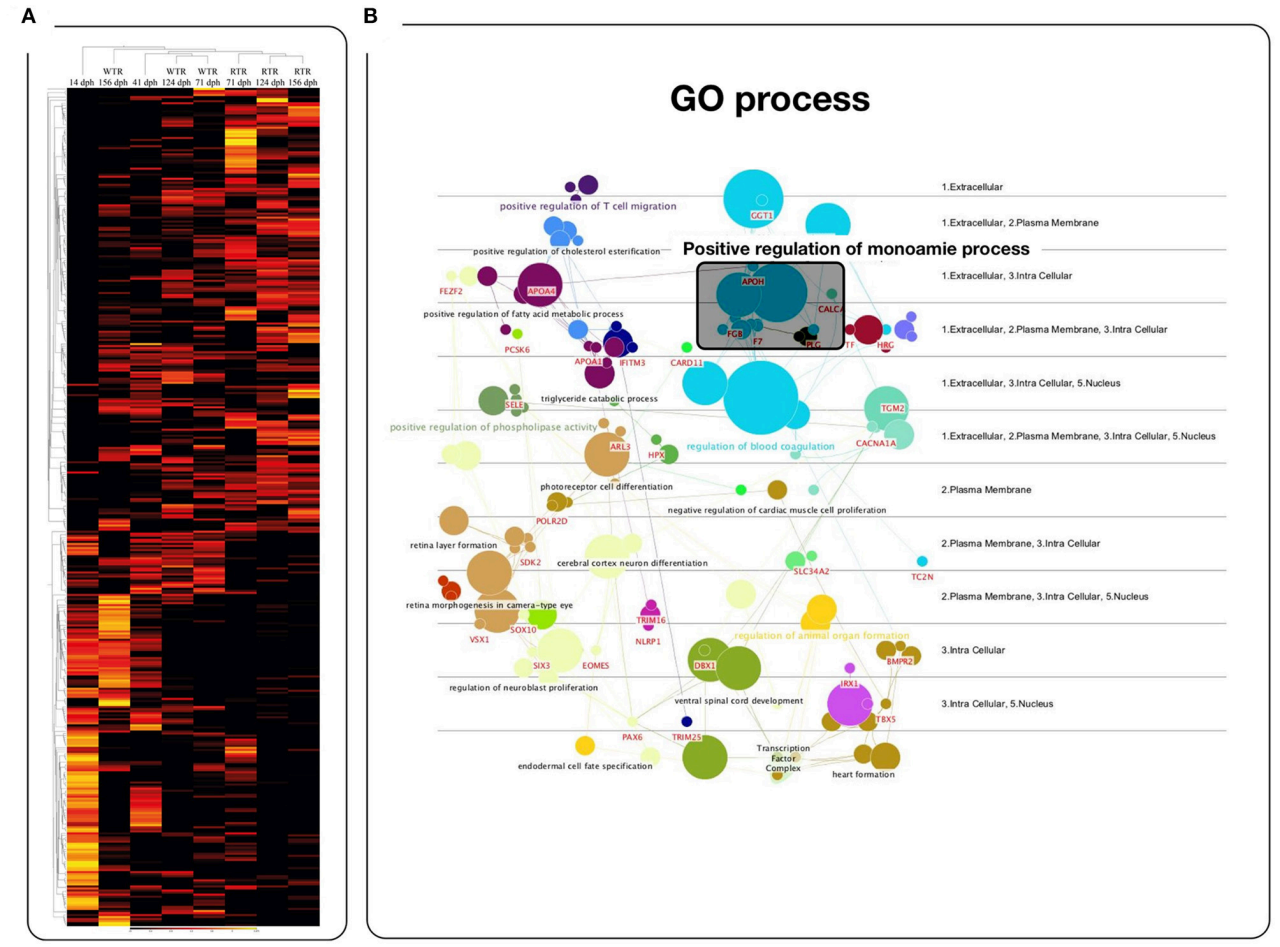
Effects of the temperature on the gene expression levels. **(A)** Heatmaps of log2-transformed gene expression levels for rank-associated genes of each thermal range during the development. **(B)** Gene ontology enrichment analysis of 415 differentially expressed transcripts (GO DAVID and ClueGo Cytoscape Plugin) in *Salmo salar* brain during development (156 days post-hatching). Experimental groups are restricted thermal range (RTR, ΔT 1.4°C) and wide thermal range (WTR, ΔT 6.8°C). Transcript abundance is represented as color scales show relative transcript expression in RPKM values.

**Figure 3:**
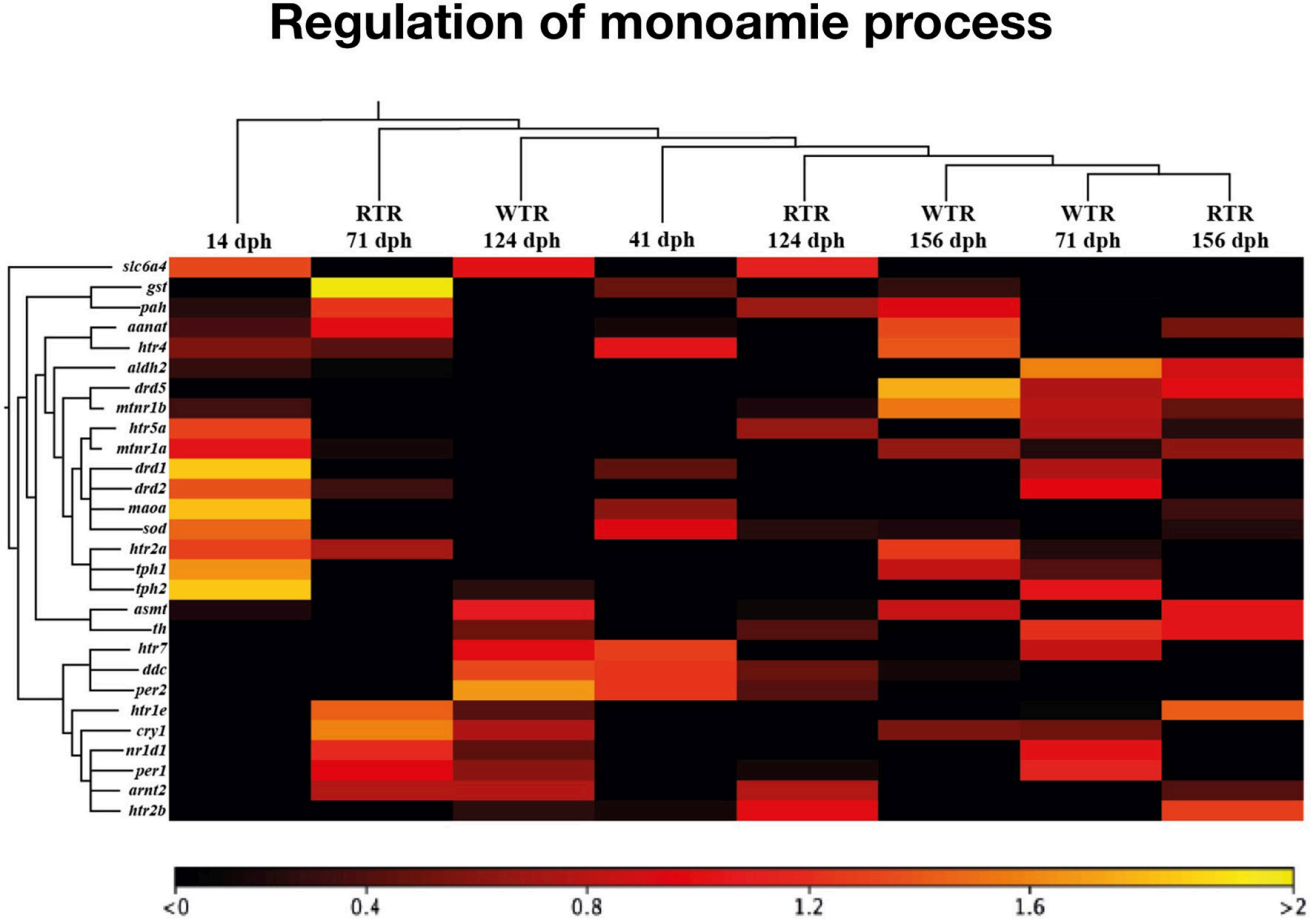
Heatmap representing the expression profile of 28 differentially expressed genes in *Salmo salar* brain during development (156 days post-hatching). Experimental groups are restricted thermal range (RTR, ΔT 1.4°C) and wide thermal range (WTR, ΔT 6.8°C). Transcript abundance is represented as color scales show relative transcript expression in RPKM values.

To explore how thermal ranges drive changes in gene regulation, we profiled gene expression using RNA-Seq in the brain of salmon under two different thermal conditions, (1) wide thermal range (WTR), and (2) restricted thermal range (RTR). A total of 415 differentially expressed genes (DEGs) were obtained (log_2_ fold change ≥ |2|; *P* ≤ 0.05). The RPKM values of selected candidate genes were visualized in a heatmap based on a hierarchical clustering of features (CLC Genomic Workbench nomenclature) (Figure 2). Statistical analysis confirmed that thermal ranges modified the transcriptomes of Atlantic salmon, evidencing the formation of clusters of transcripts with different expression patterns. Our RNA-seq results show that genes related to the biosynthesis of dopamine and serotonin, such as *dcc* (124 and 156 dph), *tph1* (71 and 156 dph) and *tph2* (71 and 124 dph) were upregulated in the group of fish with access to thermal gradient (WTR), while genes involved in the degradation of monoamines, such as *monoamine oxidase a* (*maoa*) and *aldehyde dehydrogenase 2 family* (*aldh2*) were downregulated in the same group of fish at 156 dph. Genes related to the biosynthesis of melatonin such *aanat* (156 dph), *asmt* (124 dph) and *mtnr1b* (71 and 156 dph) were upregulated in WTR. Furthermore, the expression of clock genes *per1-2, nr1d1* and *cry1* were upregulated in the WTR group and only *arnt2* was upregulated in RTR group (Figure 3).

### Absolute mRNA abundance quantification

**Figure 4:**
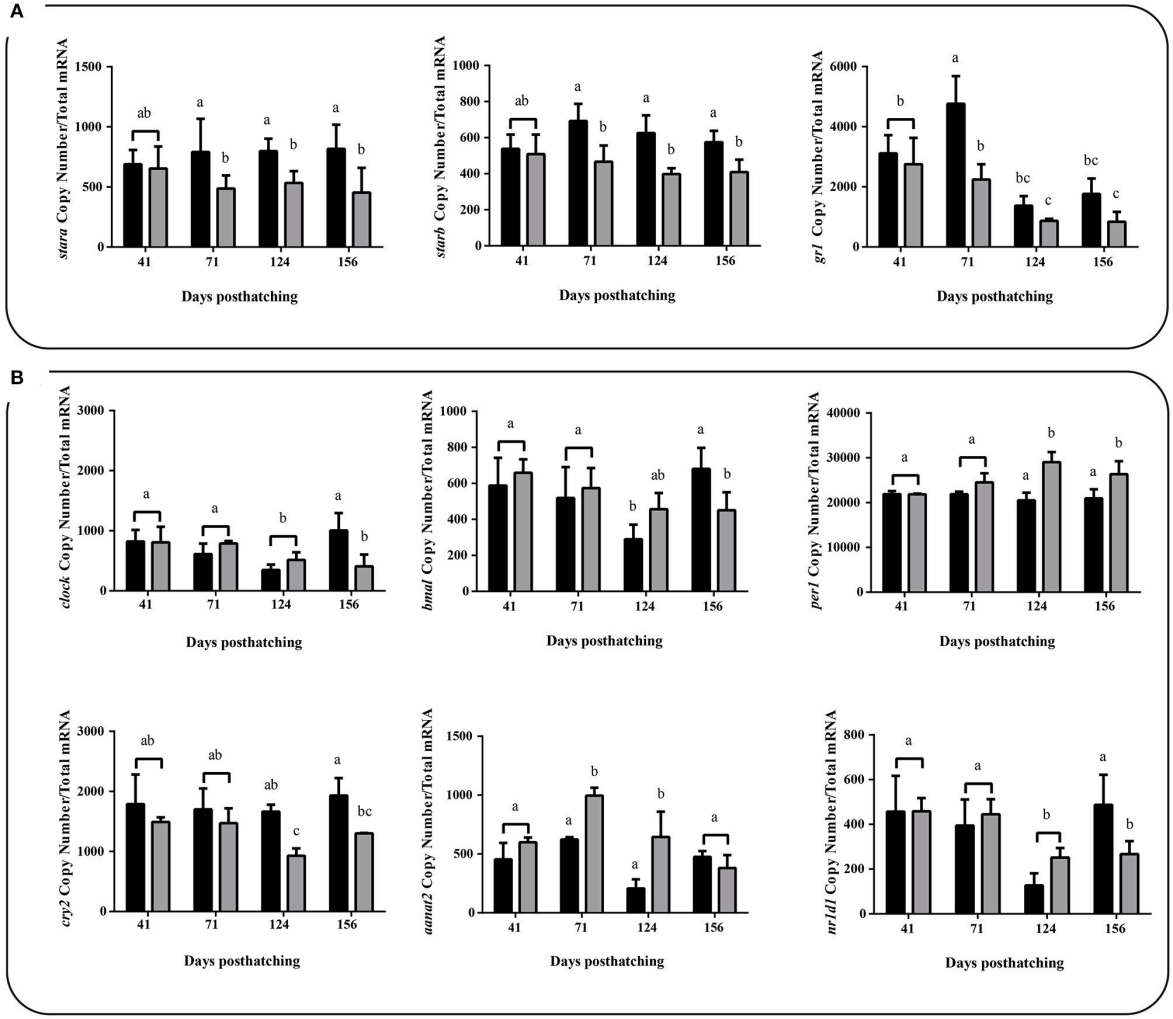
Effect of thermal range in *Salmo salar* during development (156 days post-hatching). **(A)** Liver expression profiles for *stara, starb*, and *glucocorticoid receptors 1* (*gr1*). **(B)** Brain expression profiles for clock genes. Values are represented as the mean mRNA abundance ± SD. The experimental groups are: restricted thermal range (RTR, ΔT 1.4°C; black) and wide thermal range (WTR, ΔT 6.8°C; gray). Different letters denote significantly different mRNA levels between groups (2 way ANOVA; *p* < 0.05).

We also profiled the expression of several genes related to the most commonly indicators of stress using absolute mRNA abundance quantification, which did not show large differences with the registered by the analysis of RNA-seq. Among these were cortisol biosynthesis related genes (liver samples) and clock genes (brain samples), important regulators of circadian rhythms, under two different thermal conditions (WTR and RTR). Both *stara* [two-way ANOVA; *F*_(3, 88)_ = 3.249; *p* = 0.0496] and *starb* [two-way ANOVA; *F*_(3, 88)_ = 3.334; *p* = 0.0461] showed significantly higher mRNA abundance in the restricted thermal range group (RTR) when compared to the wide thermal range group (WTR) from 71 dph onwards. *gr1* transcriptional levels were also higher in the RTR group; however, significant differences were registered only at 71 dph [two-way ANOVA; *F*_(3, 88)_ = 3.956; *p* = 0.0291, Figure 4A]. We measured absolute mRNA abundance of four clock genes (*bmal, clock, per1* and *cry2*) in both thermal groups of fish (RTR and WTR). According to Figure 4B, in wide thermal range (WTR), *per*1 (124 and 156 dph) showed a significantly higher mRNA abundance [two-way ANOVA; *F*_(3, 88)_ = 6.389; *p* = 0.0047]. While, *bmal, clock* (156 dph) and *cry2* (124 and 156 dph) transcriptional levels were higher in the RTR group [two-way ANOVA; *bmal, F*_(3, 88)_ = 4.014; *p* = 0.0263; *clock, F*_(3, 88)_ = 5.607; *p* = 0.008 and *cry2, F*_(3, 88)_ = 3.321; *p* = 0.0466]. Additionally, *aant2* showed a significantly higher mRNA abundance [two-way ANOVA; *F*_(3, 88)_ = 7.445; *p* = 0.0038] in the wide thermal range group (WTR) at 71 and 124 dph when compared to the restricted thermal range group (RTR). For detail in *p*-values for each factor and the interaction see Supplementary Table 2.

### Monoamine and melatonin content

**Figure 5:**
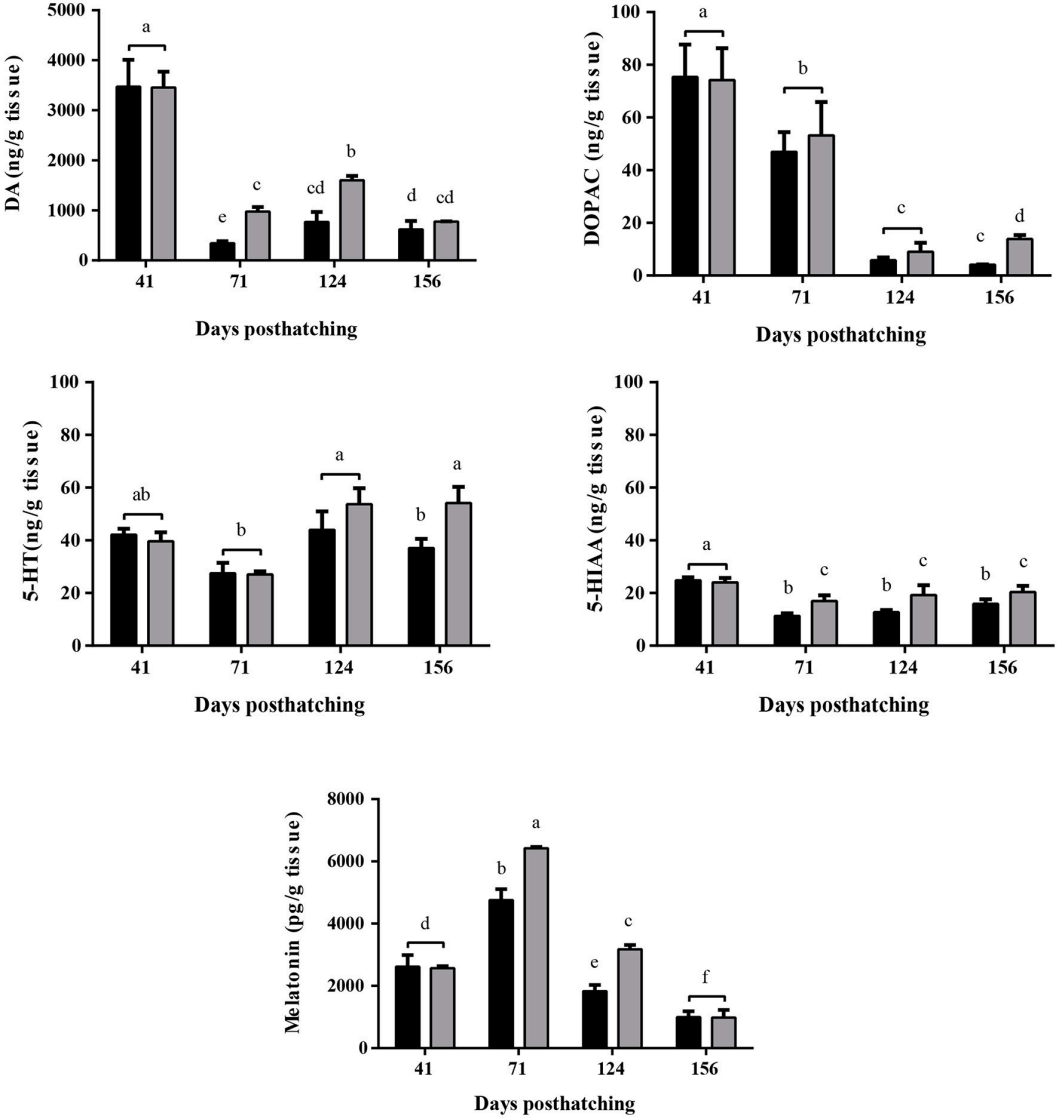
Effect of thermal range in *Salmo salar* brain serotonin (5-HT), 5-hydroxyindoleacetic acid (5-HIAA), dopamine (DA), 3,4-dihydroxyphenylacetic acid (DOPAC) and melatonin concentrations during the development (156 days post-hatching). Values are represented as the mean ± SD. The experimental groups are restricted thermal range (RTR, ΔT 1.4°C; black) and wide thermal range (WTR, ΔT 6.8°C; gray). Different letters denote significantly different concentrations between groups (2 way ANOVA; *p* < 0.05).

To inspect hormone levels, we contrasted serotonin (5-HT) content, its metabolite 5-hydroxindoleacetic acid (5-HIAA) dopamine (DA) and its intermediate metabolite, 3,4-dihydroxyphenylacetic acid (DOPAC). Finally, melatonin content in each fish under both experimental set-ups (WTR and RTR) was assesed. The monoamine profile shows statistical differences the interaction of the thermal range (WTR-RTR) and the time (Figure 5, for detail in *p-*values for each factor and the interaction see Supplementary Table 2). No significant differences were registered between brain content of 5-HT in both groups at 41, 71, and 124 dph. However, at 156 dph, 5-HT content was significantly higher in the WTR group [two-way ANOVA; *F*_(3, 64)_ = 4.210; *p* = 0.0327]. The same pattern was observed for 5-HIAA onward 71 dph [two-way ANOVA; *F*_(3, 64)_ = 3.565; *p* = 0.0444]. When assessing dopamine (DA) levels in brains of fish from RTR and WTR group, and how this level changed over time, we observed significantly higher contents of DA in the WTR group at 71 and 124 dph when compared to RTR group [two-way ANOVA; *F*_(3, 64)_ = 4.058; *p* = 0.0362]. DA metabolite, 3,4-dihydroxyphenylacetic acid (DOPAC), was statistically higher in the WTR group at 156 dph. It is important to remark that both DA and DOPAC [two-way ANOVA; *F*_(3, 64)_ = 1.670; *p* = 0.0213] showed high levels in both experimental groups at 41 dph, with a significant decrease at 71 and 124 dph for DA and DOPAC, respectively. Similar patterns for 5-HT, DA, and their respective metabolites where observed in body monoamine content in both groups (Figure 5 and Supplementary Figure 1). Similar to the monoamine content results, we also observed higher levels of melatonin in brains and body of the WTR group when compared to RTR group at 71 and 124 dph (Figure 5 and Supplementary Figure 1).

### Group structure and behavioral records

**Figure 6:**
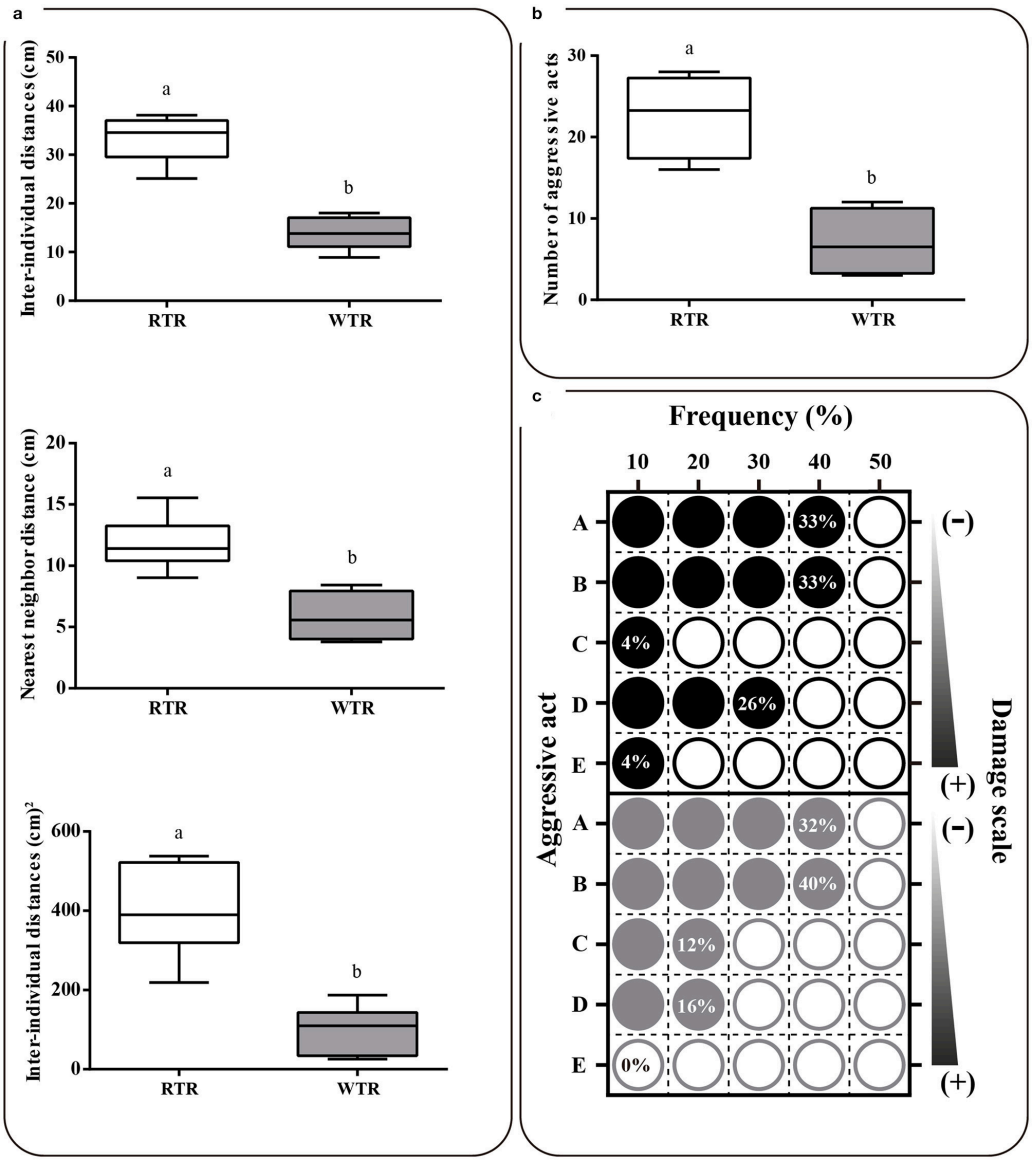
Effect of thermal range in behavioral of *Salmo salar*. **(a)** Nearest neighbor distances, inter-individual distances, and variance of interindividual distances. **(b)** Number of aggressive acts **(c)** Matrix plots registered differences in frequency (%) and potential damage of each aggressive act. Behavioral patterns observed and counted as one aggressive act were: (A) chasing without nipping or biting, (B) nipping without prior chasing, (C) biting without prior chasing, (D) chasing that ended up as nipping and (E) chasing that ended up as biting. The experimental groups are: restricted thermal range (RTR, ΔT 1.4°C) and wide thermal range (WTR, ΔT 6.8°C). Different letters denote significant differences between groups (Two-tailed Student's *t*-test; *p* < 0.05).

Group structure results show that in the wide thermal range group (WTR) the three parameters analyzed [nearest neighbor distance (NND), mean of inter-individual distances (D) and the variance of these inter-individual distances (V)] were significantly lower (Two-tailed Student's *t*-test, *p* < 0.0001), when compared to the individuals kept in a restricted thermal range (RTR), so that NND, D and V decreased around 50, 58, and 75%, respectively (Figure 6a). Statistical differences were observed between RTR and WTR groups regarding the number of aggressive encounters registered (Two-tailed Student's *t*-test, *p* < 0.0001; Figure 6b, Supplementary Table 2). Regarding behavior, in the RTR group we observed a mean of 23 aggressive encounters in the 10 min of video recording. In contrast, a significantly lower number of aggressive encounters was registered for the WTR group, with a mean of 7 encounters. Most aggressive encounters in the RTR group corresponded to chasing without nipping or biting, nipping without prior chasing and chasing that ended up as nipping, representing the 92% of all aggressive encounters (Figure 6c).

### Growth performance, thermal preferendum, and Fin damage

**Figure 7:**
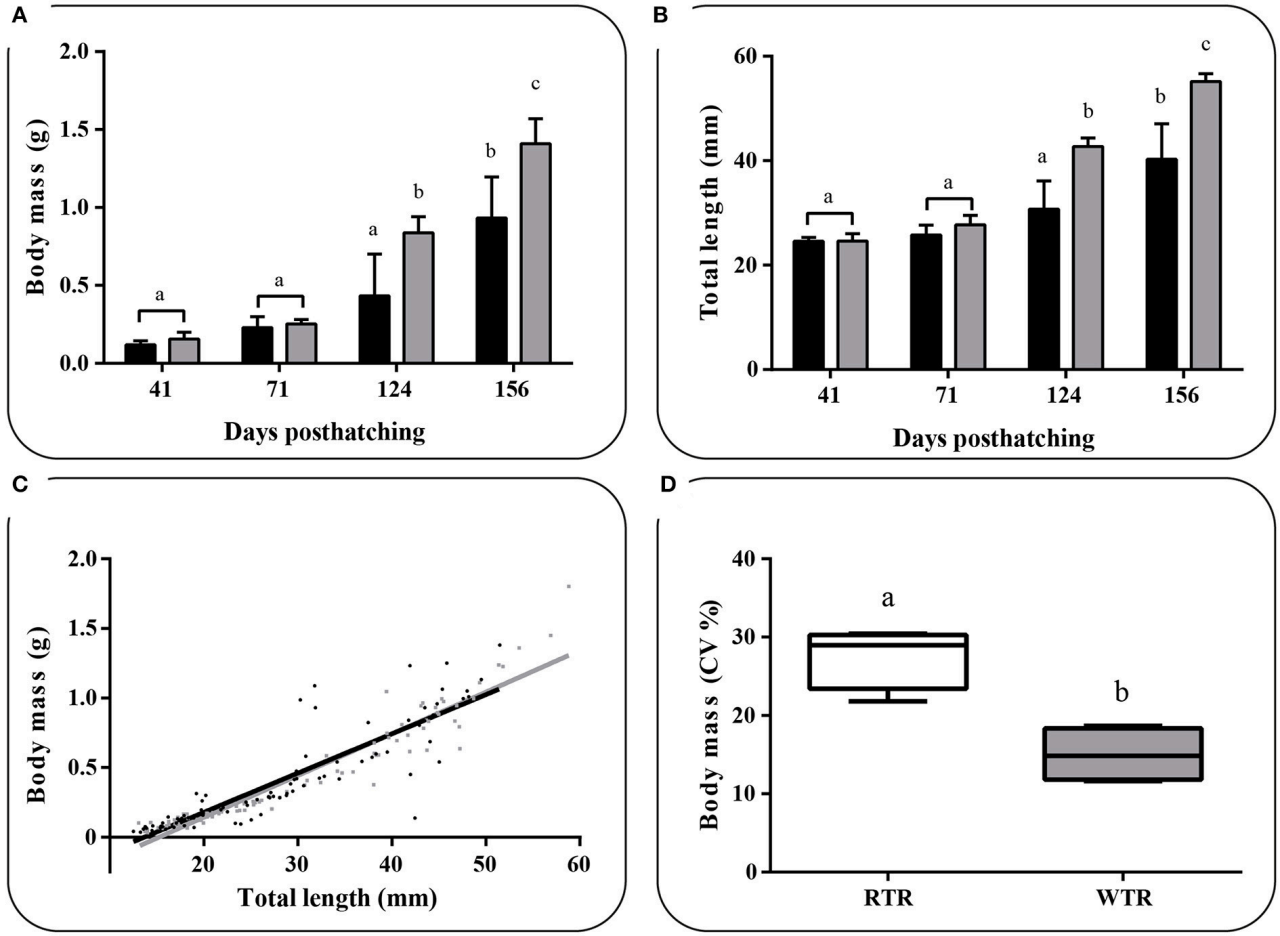
Effect of thermal range in *Salmo salar* growth during the development (156 days post-hatching), expressed as **(A)** changes in body weight, **(B)** changes in body total length, **(C)** weight/length relationship and **(D)** Box and whiskers plots registered differences in coefficients of variation for body weight. Values are represented as the mean ± SD. The experimental groups were: restricted thermal range (RTR, ΔT 1.4°C; black) and wide thermal range (WTR, ΔT 6.8°C; gray). Different letters denote significant differences between groups (2 way ANOVA; Two-tailed Student's *t*-test; *p* < 0.05).

**Figure 8:**
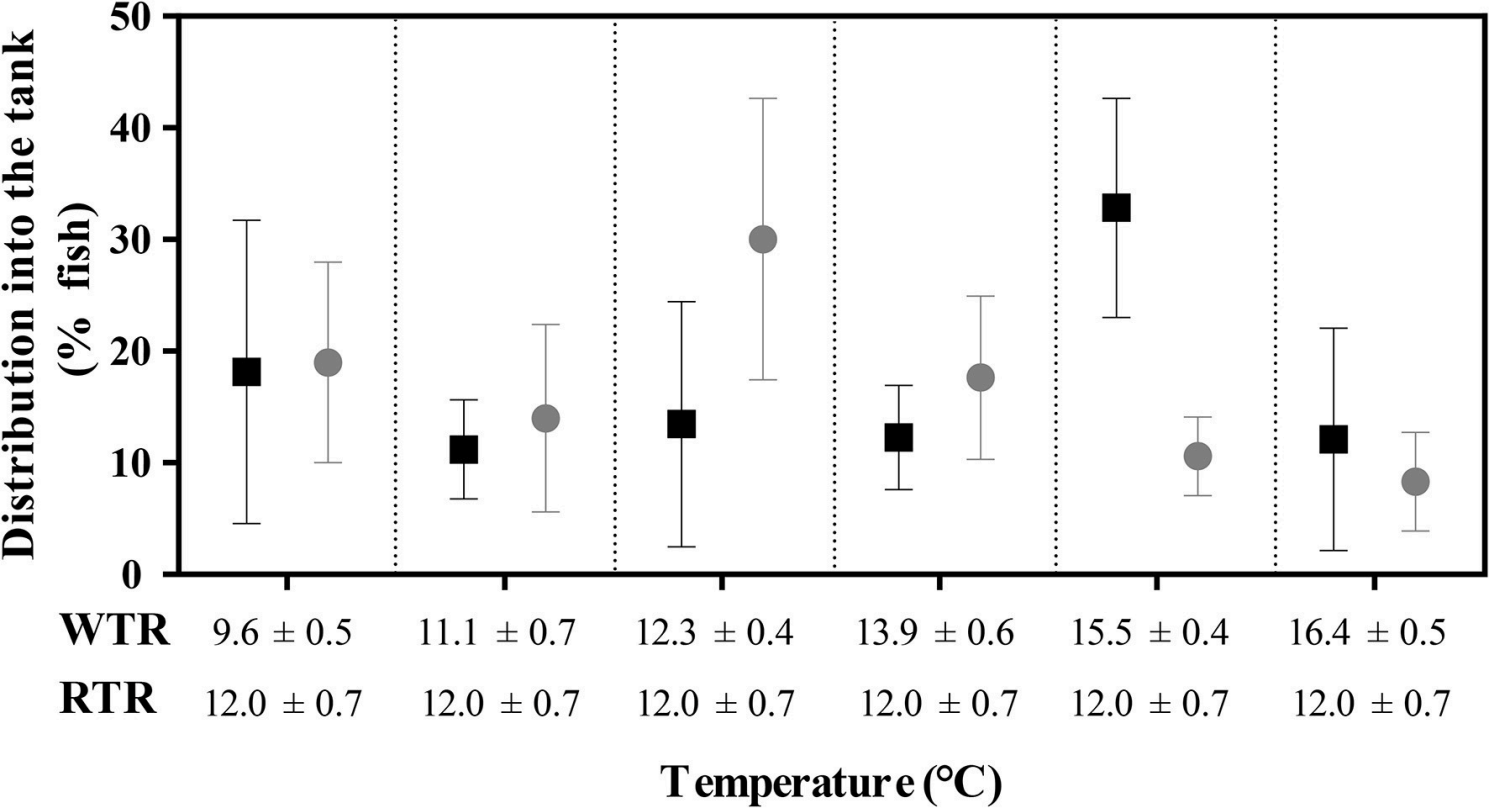
Effect of thermal range in the final thermal preferendum of *Salmo salar*. Values are represented as the mean ± SD. The experimental groups are restricted thermal range (RTR; black) and wide thermal range (WTR; gray). Different letters denote significantly differents between groups (2 way ANOVA; *p* < 0.05).

**Figure 9:**
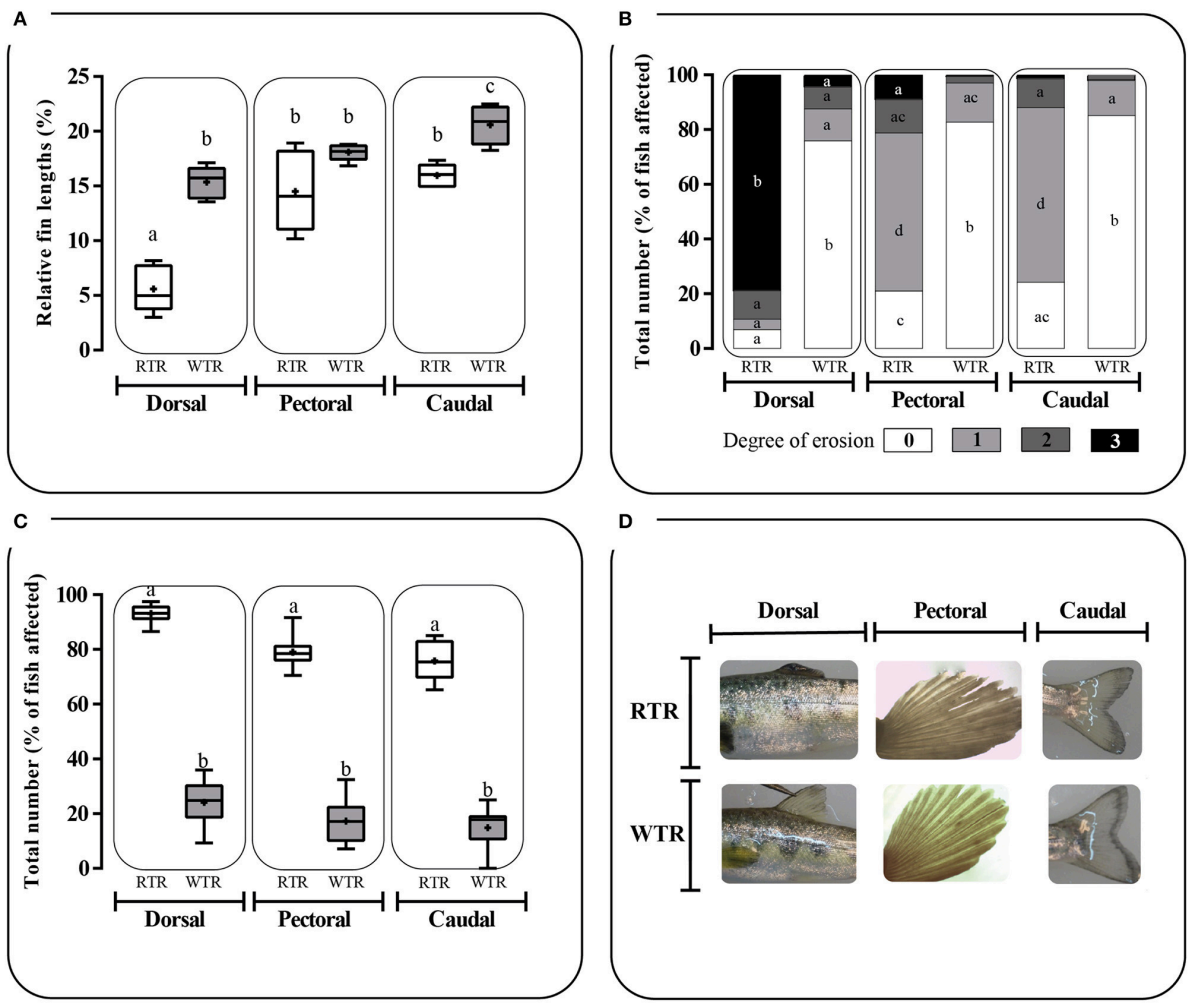
Effect of thermal range in *Salmo salar* fin damage expressed as: **(A)** Relative Fin Index, **(B)** degree of fin erosion and **(C)** total number of fish affected in **(D)** dorsal, pectoral and caudal fin. Fin erosion was measured utilizing an ordinal scale of 0, 1, 2, and 3, corresponding to no erosion (0% of fin eroded), mild erosion (1–24% of fin eroded), moderate (25–49% of fin eroded) and severe erosion (>50% of fin eroded), respectively. Values are represented as the mean ± SD. The experimental groups are: restricted thermal range (RTR, ΔT 1.4°C; black) and wide thermal range (WTR, ΔT 6.8°C; gray). Different letters denote significant differences between groups (2 way ANOVA; *p* < 0.05).

Individuals kept in a restricted thermal range (RTR) and fish allowed to move freely in a wide thermal range (WTR) showed a mean Fulton's condition factor (K) of 1,10 ± 0,23, and 1,09 ± 0,27, respectively (Table 1). Regarding the K index, as shown in Figure 7C, no significant differences were registered for fish growth, indicating that individuals from both groups had a proportional weight, in accordance to their respective body lengths [two-way ANOVA; *F*_(3, 16)_ = 1.180; *p* = 0.349; slope *F*_(1, 172)_ = 0.91034, *p* = 0.3414 and intercept *F*_(1, 173)_ = 0.779304, *p* = 0.3786, Supplementary Table 2]. In addition, when analysing growth trajectories, we observed that only the interaction between thermal range (WTR) and development time display significant differences when assesing both length and body mass [two-way ANOVA; *F*_(3, 232)_ = 72.06; *p* < 0.0001 and *F*_(3, 232)_ = 37.00; *p* < 0.0001 respectively, Figures 7A,B, for detail in *p*-values for each factor and the interaction see Supplementary Table 2]. We also calculated the coefficient of variation (CV) of weight in each thermal group. Interindividual variance of the RTR group was significantly higher when compared to WTR group (Two-tailed Student's *t*-test, *p* < 0.0001; Figure 7D). In addition, we observed considerable differences when comparing individuals kept in a restricted thermal range (RTR) and fish allowed to move freely in a wide thermal range (WTR) in parameters related to welfare. The behavioral analysis of the thermal preferendum (Figure 8) highlights that individuals placed in the thermal gradient tank constantly moved through the tank and did not prefer a specific zone (no statistical differences shown, two-way ANOVA Supplementary Table 2). These results suggest that the observed differences are not related with the fact that WTR fish spent more time in the warmer water layer in the tank. In concordance with the aforementioned results, fin damage analysis shows that although both RTR and WTR individuals display some level of body damage, the number of affected individuals was significantly higher in the RTR group, with around 80% of individuals affected for all dorsal, pectoral and caudal fins (Figure 9). In contrast, WTR affected individuals did not exceed the 40%, with no significant differences between dorsal, pectoral and caudal fin [two-way ANOVA; *F*_(2, 174)_ = 6.391; *p* = 0.0021; Figures 9C,D]. The RFI index was normalized dividing the maximum total fin length (longest fin-ray length from the body) by the fork length in each individual fish. The results observed in the RFI index show significant differences in the dorsal and caudal fins, being higher in the WTR individuals when compared to the RTR ones [two-way ANOVA; *F*_(2, 174)_ = 39.98; *p* < 0.0001; Figure 9A]. As previously described, we assessed the degree of fin erosion using an ordinal scale. For all dorsal, pectoral and caudal fins, WTR groups showed approximately 80% of individuals with no erosion. In contrast, only a few individuals showed this degree in the RTR group. Significant erosion (degree 3) was observed in dorsal fins of the RTR group, with more than 80% of the individuals affected. In the same group, pectoral and caudal fin erosion was lower, with most individuals showing mild erosion (degree 1) [two-way ANOVA; *F*_(15, 696)_ = 9952; *p* < 0.0001, Figure 9B, for detail in *p*-values for each factor and the interaction see Supplementary Table 2].

Table 1Effect of thermal range in *Salmo salar* Fulton's condition factor.

**Table:** 

**Fulton's condition factor**	**RTR ($\bar{\text{X}}$ ± SD)**	**WTR ($\bar{\text{X}}$ ± SD)**
41 dph	0.808 ± 0.162	1.090 ± 0.502
71 dph	1.311 ± 0.253	1.196 ± 0.242
124 dph	1.158 ± 0.088	1.129 ± 0.045
156 dph	1.129 ± 0.109	0.935 ± 0.138

**Table:** 

**ANOVA table**	**SS**	**DF**	**MS**	**F (DFn, DFd)**	***p*****-value**
Thermal treatment ^*^ dph	0.195	3	0.065	*F*_(3, 16)_ = 1.180	0.349
dph	0.317	3	0.106	*F*_(3, 16)_ = 1.914	0.168
Thermal treatment	0.001	1	0.001	*F*_(1, 16)_ = 0.021	0.886
Residual	0.883	16	0.055		

*Values are represented as the mean ± SD. The experimental groups are: restricted thermal range (RTR, ΔT 1.4°C) and wide thermal range (WTR, ΔT 6.8°C). Different letters denote significant differences between groups (2way ANOVA; p < 0.05)*.

## Discussion

Fish are reared in captivity for a number of different reasons, for example, food production, conservation, stock enhancement, angling, ornamental purposes and research (3). Life in captivity generally promotes maladaptive or unwanted traits compared to those animals adapted to wild conditions (39, 40). Fish farmers and researchers have discussed the mechanisms and methods able to avoid the occurrence of unappropriated characteristics in captive fish, especially when they are destined for release into nature (41), but also when fish are used as model organisms in laboratories (42). In addition, there is a rising public concern for the welfare of captive fish and their difficulty to develop natural behaviors (43). Fish welfare has been defined as the quality of life experienced by the animal itself, such experience is seen as the animal's qualitative assessment of fulfillment of their welfare needs (3, 44). Frequently, fins damage and higher growth rates (Fulton index) are increasingly being used as potential indicators of the welfare of fish (16, 45). While several aspects of fish welfare have received significant attention in the last decades, the question whether the thermal enrichment under captivity conditions impact on the stress and welfare is scarcely discussed in scientific literature. Our results imply, that introducing the thermal variation on the containment unit impact significantly on the hormone performance, allowing preferred behaviors and give animals the possibility to choose their environment. The present results suggest that fish kept at a restricted thermal environment display severe fin damage in terms of relative length and degree of erosion. In both thermal environments, fish moved freely through the tank and freely choose a temperature layer. This results are supported by the video records and data illustrated in Figure 9 and suggest that the robust differences observed in the growth trajectories, hormone and molecular performance were due tothe thermal enrichment provided in the WTR tank and not due to a preference for a specific zone in the tank. Previous studies have reported that temperature can considerably affect fish growth (17), the present results propose that fish kept at a WTR show higher standards when compared to RTR ones, as far as body condition is concerned. Considering the present context, the aim of the current study was to investigate the degree of influence that spatial variation in temperature range has on *S. salar* welfare, stress or molecular underlying mechanisms. We hypothesized that fish response to spatially variable temperatures would involve temperature-dependent effects related to metabolic demands, such as growth, and hormone performance during development. Understanding the potential effects for this “bottom-up” interaction of thermoregulatory behavior with major biological processes is relevant for identifying reactions to dissimilar thermoregulatory ranges, particularly in the larger context of environmental enrichments of aquaculture or captivity conditions.

The growth and the coefficient of variation (CV) is a parameter often impacted by temperature, a phenomenon known as growth depensation and defined as the increase in size distribution variance over time due to differential growth rates (46). In captivity, the growth depensation induce those which grow poorly to be socially subordinate (47). In the present study, through our novel experimental set-up we were able to determine key parameters associated with the establishment of social structure which might illustrate changes in the frequency of dominance/subordination interactions induced by the thermal environment (48). Similar results have been observed and show that deficient thermal environments affect inter-individual relations, promoting territorial and aggressive behaviors and consequently compromise fish welfare (31, 49). The present data might explain the growth depensation as a result of a higher frequency of dominance/subordination interactions, however, further studies will be required to fully test this hypothesis.

In culture or captivity conditions, the means to improve the welfare conditions might consist of choice the best species which are more amenable to a life in captivity, for example regarding aggressive behavior. The present study hypothesizes that the thermal enrichment is a key mechanism able to increase the welfare and decrease the aggressive behavior. The mechanism by which the temperature influence the stress and welfare have not been established, in contrast the physiological and molecular bases for aggressiveness have been widely revised [e.g., Winberg et al. (34); Lepage et al. (50); Johnsson et al. (9); Beiderbeck et al. (51); Dahlbom et al. (6); Mogavero et al. (13); Winberg and Thörnqvist (52)]. Most of these studies suggest that neurotransmitters like serotonin, dopamine and melatonin hormone play a central role in regulating behavior, including aggressiveness or hyperactivity. The collective data suggest that the decreased level of dopamine and serotonin is correlated with an increase of aggressiveness or hyperactivity (12, 53). Our data are in concordance with the previous studies and show that serotonin, and the dopamine were significantly lower in the fish housing in thermal restricted environments (RTR) exhibiting more aggressive encounters and social conflicts.

Previous reports also have established the role clock related genes such as *per1-2, bmal1, cry1-2*, and *clock* in the modulation of monoamine oxidase a (MAOA), a key enzyme involved in the degradation of monoamines including dopamine or serotonin tightly linked with the regulation of aggressive behavior (54, 55). Specifically, it has been determined that a reduced gene expression of *per1* decreased the levels of dopamine (12). Regarding circadian rhythm control in the suprachiasmatic nucleus, synchronization is induced by serotonin activation, that is directly linked to the melatonin production (56). Our analysis show that the mRNAs linked with the circadian clock were juxtaposed regulated by both thermal environments. We registered a peak of both melatonin hormone and *per1* mRNA abundance in individuals rearing in environmental thermal enrichment (57). In mammals, *per1* mRNA also acts as a negative regulator of *clock* and *bmal* genes (57, 58), which might be explain the observed pattern of fish rearing in restricted thermal environments. This group of individuals (RTR) showed higher expression of *clock* and *bmal* in contrast to WTR ones, however, additional investigation is required as post-translation modifications may occur. Comparison of data sets obtained from RNA-Seq and Absolute-PCRs using the same set of samples showed a good correlation between gene expression profiles. However, some authors suggested that RNA-Seq had the best performance detecting low abundance transcripts or detecting isoforms that allow the identification of genetic variants (59). The performance difference between in the present work between both tools is an area of controversy in the scientific community. PCRs could use internal controls in order to obtain a high reproducibility when analyzing expression data by facilitating a choice between many types of transformation/normalization methods i.e., Efficiency Analysis informs us which methods to choose.

The responsiveness of the HPI-axis was also impacted by temperature. We observed that thermal enrichment provide a complex environment, which allows maximizing normal behaviors and minimizing stress-induced behaviors under captivity conditions (60, 61). Cholesterol is a rate-limiting metabolite as it is the source substrate for cortisol production, and *steroid acute regulatory protein* (*star*) plays the crucial role of transferring the hydrophobic cholesterol, across the aqueous barrier between the outer and inner mitochondrial membrane (62, 63). In cow, it has been demonstrated that mRNA abundance of *star* mirrors cortisol plasmatic levels (64, 65), and in salmon similar results have been observed during chronic stress (63, 66, 67). The present results show that fish under a restricted thermal range (RTR) are able to trigger the expression of mRNAs as *stara* and *starb* (68–70), suggesting that it can be associated to stress condition in contrast to the observed in wide thermal range fish (WTR), however additional experiments including plasmatic cortisol measures should be needed to robustly test this hypothesis. Regarding the glucocorticoid receptor (*gr*), an important mediator of the stress response at the tissue level, has been observed that salmonid fish have duplicate amino acid sequences “g*r1* and *gr2*” (71–73). The differential cortisol receptor subtype signaling may be defined by the kind of stressor (74, 75), tissue distribution and co-localization (71). In our experiment, the decrease observed for *gr1* mRNA abundance suggests a need for decreased cortisol signaling during development in the restricted thermal environment. This negative feedback of *gr1* gene expression has been previously observed in salmon, triggered by elevated plasma cortisol levels during chronical stress condition (70, 72, 76–79), but further analysis will be addressed to test this hypothesis.

In summary, our determinations that the spatial variation of temperature which never has been considered as an environmental/occupational enrichment is a key component to drive an increase the welfare relative to the reduction of physical and psychological monotony. The present results show that the thermal enrichment allows the induction of deep alterations in gene transcription of circadian-clock related genes as well as the modulation of hormone levels that drive a decrease in aggressiveness and territoriality. Our results highlight that the thermal range amplitude might influence health, stress and welfare condition of fish under farm conditions, thus making this study important for any aquaculture researcher, stakeholders and policy makers. The reported results should be contributing novel insights to a central question in endocrinology, and behavioral ecology, where spatial variation of temperature directly impact on the hormonal performance, growth and metabolism and finally on the welfare, consequently, researchers studying adaptation, climate change, behavioral ecology and immunology will be interested in this general phenomenon.

## Author contributions

The study was conceived by SB. The behavioral experiments have been performed by NS and AA. JM analyzed the hormone data. RF analyzed the RNA-seq data. LT, AD, NS, BC, and JV have performed the gene expression analysis and provided extensive additional input. SB provided the funding acquisition. SB and LT drafted the manuscript with substantial contributions from all other authors.

### Conflict of interest statement

The authors declare that the research was conducted in the absence of any commercial or financial relationships that could be construed as a potential conflict of interest.
