# Supplementary material for: Thermal Modulation of Monoamine Levels Influence Fish Stress and Welfare
Source: Front Endocrinol (Lausanne). 2018 Dec 3;9:717. doi: 10.3389/fendo.2018.00717 (PMC6287116; doi:10.3389/fendo.2018.00717)
Supplement: Supplementary file 1 [file Data_Sheet_1.docx]

**Thermal modulation of monoamine levels influence the fish stress and welfare**

Nataly Sanhueza^1†^, Andrea Donoso^1†^, Andrea Aguilar^1^, Rodolfo Farlora^2^, Beatriz Carnicero^1^, Jesús Manuel Míguez^3^, Lluis Tort^4^, Juan Antonio Valdes^5^, Sebastian Boltana^1*^

^1^*Interdisciplinary Center for Aquaculture Research (INCAR), Department of Oceanography, Biotechnology Center, University of Concepción,* *4030000 Concepción, Chile.*

^2^*Instituto de Biología, Facultad de Ciencias Universidad de Valparaíso, Chile.*

^3^*Laboratorio de Fisioloxía Animal, Departamento de Bioloxía Funcional e Ciencias da Saúde, Facultade de Bioloxía, Universidade de Vigo, Spain.*

*^4^Department of Cell Biology, Physiology and Immunology, Universitat Autonoma de Barcelona, 08193 Barcelona, Spain*

*^5^Universidad Andrés Bello, Facultad de Ciencias Biológicas, Santiago, Chile*

# Supplementary Figures and Tables

## Supplementary Figures

**
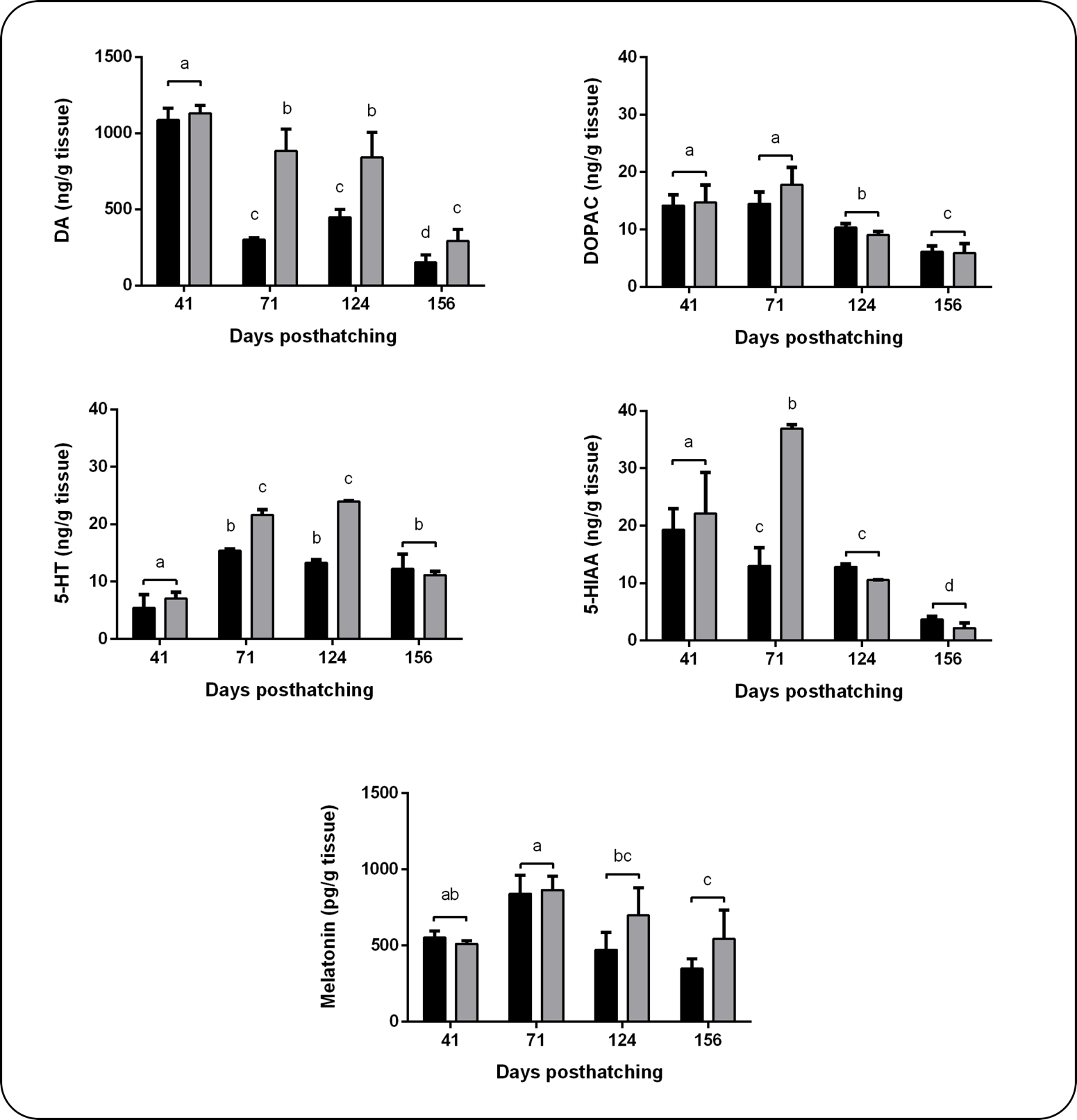
**

**Supplementary Figure 1.** Effect of thermal range in *Salmo salar* body serotonin (5-HT), 5-hydroxyindoleacetic acid (5-HIAA), dopamine (DA), 3,4-dihydroxyphenylacetic acid (DOPAC) and melatonin concentrations during the development (156 days post-hatching). Values are represented as the mean ± SD. The experimental group are: restricted thermal range (RTR, ΔT 1.4 °C; black) and wide thermal range (WTR, ΔT 6.4°C; grey). Different letters denote significantly different concentrations between groups (2way ANOVA; P < 0.05).

## Supplementary Table

**Supplementary Table 1** mRNA primer sequences used for absolute RT-qPCR analysis

| **Primer name** | **Gene** | **Sequence 5'-3'** | **Size (bp)** | **E**  **(%)** | **Tm**  **(°C)** | **GenBank accession n°** |
| --- | --- | --- | --- | --- | --- | --- |
| *clock* | *clock circadian regulator* | F:AGAAATGCCTGCACAGTCGGAGTC | 197 | 91.3 | 64 | CA 038738 |
|  |  | R: CCACCAGGTCAGAAGGAAGATGTT |  |  |  |  |
| *per1* | *period circadian clock 1* | F: AGGGGGTCATGCGGAAGGGGAAGT | 150 | 62.35 | 66 | Unpublished |
|  |  | R: TGGGCCACCTGCATGGGCTCTGT |  |  |  |  |
| *bmal* | *aryl hydrocarbon receptor nuclear translocator like* | F: GCCTACTTGCAACGCTATGTCC | 90 | 101.2 | 66 | DY 735402 |
|  |  | R: GCTGCGCCTCGTAATGTCTTCA |  |  |  |  |
| *cry2* | *cryptochrome circadian clock 2* | F: GAGGGCATGAAGGTGTTTGAGGAG | 108 | 59.5 | 60 | DY7301105 |
|  |  | R: GTGGAAGAACTGCTGGAAGAAGGA |  |  |  |  |
| *aanta2* | *aryl hydrocarbon receptor nuclear translocator like 2* | F: GCTCTCCCTGGGCTGGTTTGAAG | 132 | 68.6 | 62 | NM_001124257.1 |
|  |  | R: CATGGATGTGCACTGCCGAGGTT |  |  |  |  |
| *nr1d1* | *nuclear receptor subfamily 1 group d member 1* | F: CCCCCAAGACGAACCCAACAAGAC | 194 | 67.5 | 62 | 1714461 |
|  |  | R: AGAGGGAGGCAAAGCGCACCATTA |  |  |  |  |
| *stara* | *steroidogenic acute regulatory* | F: CATGGAGCAGATGGGAGATT | 154 | 111.5 | 62 | DQ415678.1 |
|  |  | R: CTTGGCACAGCGAACACTAA |  |  |  |  |
| *starb* | *steroidogenic acute regulatory* | F: AGGATGGATGGACCACTGAG | 161 | 111 | 64 | XM_014171084.1 |
|  |  | R: GTCTCCCATCTGCTCCATGT |  |  |  |  |
| *gr* | *glucocorticoid receptor (nr3c1)* | F: AGAAGCCTGTTTTTGGCCTGTA | 101 | 127 | 61 | NM_001124730.1 |
|  |  | R: AGATGAGCTCGACATCCCTGAT |  |  |  |  |
